# Acoustofluidics for simultaneous nanoparticle-based drug loading and exosome encapsulation

**DOI:** 10.1038/s41378-022-00374-2

**Published:** 2022-04-28

**Authors:** Zeyu Wang, Joseph Rich, Nanjing Hao, Yuyang Gu, Chuyi Chen, Shujie Yang, Peiran Zhang, Tony Jun Huang

**Affiliations:** 1 Department of Mechanical Engineering and Materials Science, Duke University, Durham, NC 27708 USA; 2 Department of Biomedical Engineering, Duke University, Durham, NC 27708 USA

**Keywords:** Microfluidics, Nanofabrication and nanopatterning

## Abstract

Nanocarrier and exosome encapsulation has been found to significantly increase the efficacy of targeted drug delivery while also minimizing unwanted side effects. However, the development of exosome-encapsulated drug nanocarriers is limited by low drug loading efficiencies and/or complex, time-consuming drug loading processes. Herein, we have developed an acoustofluidic device that simultaneously performs both drug loading and exosome encapsulation. By synergistically leveraging the acoustic radiation force, acoustic microstreaming, and shear stresses in a rotating droplet, the concentration, and fusion of exosomes, drugs, and porous silica nanoparticles is achieved. The final product consists of drug-loaded silica nanocarriers that are encased within an exosomal membrane. The drug loading efficiency is significantly improved, with nearly 30% of the free drug (e.g., doxorubicin) molecules loaded into the nanocarriers. Furthermore, this acoustofluidic drug loading system circumvents the need for complex chemical modification, allowing drug loading and encapsulation to be completed within a matter of minutes. These exosome-encapsulated nanocarriers exhibit excellent efficiency in intracellular transport and are capable of significantly inhibiting tumor cell proliferation. By utilizing physical forces to rapidly generate hybrid nanocarriers, this acoustofluidic drug loading platform wields the potential to significantly impact innovation in both drug delivery research and applications.

## Introduction

Nanoscale drug delivery systems have gained prominence in the past decade due to the promise of novel therapeutic applications^1,2^. By loading drug molecules into nanoscale carriers, controllable release and active targeting can be achieved^3^. Compared to small drug molecules in suspension, nanoparticles demonstrate enhanced permeability and retention, leading to higher drug accumulation within the target cells^4,5^. Currently, most existing nanocarriers utilize porous, inert nanoparticles as the carrier; drug molecules are then diffusively loaded into the porous space^6,7^. The greatest challenge of inert nanocarriers is their tendency to have low drug loading efficiencies (<10%)^8^.

To increase the drug loading efficiency of inert nanocarriers, one promising strategy utilizes natural nanoparticles and exosomes. Exosomes are approximately 30–150 nm diameter cell-derived nanovesicles that carry genetic material, proteins, and other molecules from their cells of origin^9^. These vesicles have shown great potential for improving the drug delivery efficacy of drugs and nanoparticles^10^. With their characteristic phospholipid bilayer structure, similar to that of liposomes, exosomes can encapsulate both drugs and nanoparticles while prohibiting the release and degradation of the loaded drugs. Furthermore, compared to liposomes, exosomes are naturally produced by cells, have higher biocompatibility, and avoid clearance by the immune system^11^. Another advantage of using exosomes in drug delivery is their targeted delivery potential. Because exosomes exhibit intercellular communication *via* exocytosis and endocytosis pathways, they are natural carriers capable of delivering proteins and nucleic acids among cells^12^. Depending on the type of cell secretions, exosomes can be absorbed into specific cell populations and subsequently participate in various critical biofunctions including cell expansion associated with neuron degeneration, the proliferation and invasion of tumor cells, immune system activation, and tissue regeneration^13,14^. Previous studies have demonstrated that exosome-encapsulated silica nanoparticles have an advantageous low immune clearance combined with improved targeted delivery abilities^15^. Current exosome-encapsulated nanoparticle methods include passive incubation, freeze/thaw cycling, surface conjugation, extrusion, electroporation, and sonication^16,17^. However, these exosome encapsulation methods often have low drug loading efficiencies and/or complex, slow drug loading processes.

Here, we have developed a drug loading method based on acoustofluidics, i.e., the fusion of acoustics and fluid mechanics^18–24^, that can simultaneously concentrate drug molecules with silica nanoparticles and encapsulate the particles within exosomes, all within a single-step process. Acoustofluidics have been demonstrated to have great potential for biological particle manipulation and enrichment^25–31^, which could be applied to vesicle separation and cell patterning for diagnostic purposes^32–38^. Since acoustofluidics has the ability to manipulate nanoscale particles that contain both extracellular vesicles and nanocarriers^39–41^, this technology also has therapeutic potential including nanoparticle assembly and controllable drug release^42–47^. Surface acoustic wave (SAW)-derived droplet rotation has shown great potential in particle enrichment^48,49^. One of our recent works demonstrated the manipulation and enrichment of nanoparticles smaller than 100 nm within droplets by utilizing SAW-derived rotation and droplet resonance^50^. This SAW-derived rotation and droplet resonance mechanism is the basis of the drug loading application in this article. Based on investigations of the potential of acoustofluidics for constructing new drug nanocarriers, our device demonstrates a simple method with high drug loading efficiency and high speed. The device achieves stable drug loading efficiency using silica nanoparticles with different shapes and sizes. This versatility suggests that the acoustofluidic method has significant potential in loading and encapsulating a variety of nanocarriers and drugs into exosomes due to the lack of restrictions in terms of morphology and size. Since this acoustofluidic drug loading and exosome encapsulation method relies on physical forces that load and encapsulate nanoparticles independent of morphology and size, this platform exhibits versatility for loading and encapsulating a variety of nanocarriers and drugs into exosomes^51^. Acoustofluidic drug loading and carrier encapsulation achieved a loading efficiency of ~30% within 2 min, compared to the 0.08% loading efficiency achieved after 22 h of passive incubation^15^. The acoustofluidic drug loading device achieves high-efficiency drug loading encapsulation and simplifies the two-step process (i.e., drug loading and exosome encapsulation) into one step with significantly decreased processing time (i.e., hours to minutes). These features exhibit the potential for developing novel hybrid drug carriers that combine the advantages of both nanocarriers and exosome drug delivery platforms.

## Results

### Working mechanism

**Fig. 1:**
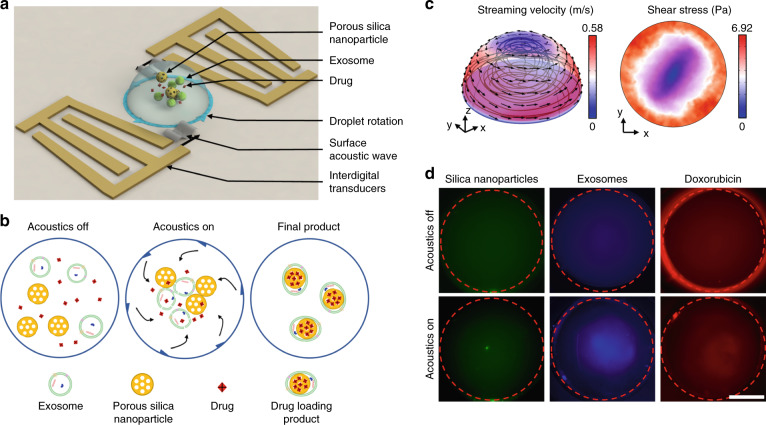
Schematics and mechanism of acoustofluidic drug loading. **a** In the acoustofluidic device, a pair of slanted interdigital transducers generate surface acoustic waves and induce droplet rotation as well as vortex streaming, which lead to concentration and fusion of the porous silica nanoparticles, exosomes, and drug within the droplet. **b** When the acoustofluidic device is off, silica nanoparticles, exosomes, and drug (e.g., doxorubicin) are uniformly distributed within the droplet. When the acoustofluidic device is activated, these particles (i.e., silica nanoparticles, exosomes, and drugs) are concentrated in the center of the droplet. The high concentration and shear stress simultaneously induce both drug loading into the silica nanoparticles and encapsulation of the silica particles within the exosomes. **c** Numerical simulation showing acoustic microstreaming and induced shear stress distribution when the device is activated. The flow direction is toward the center of the droplet. **d** Fluorescent images of the silica nanoparticles, exosomes, and doxorubicin show different levels of concentration before and after the acoustofluidic device is activated. Scale bar: 1 mm.

The mechanism of the acoustofluidic-derived droplet rotation and derived microstreaming and shear rate pattern was established in our previous work^50^. As a brief description, a pair of slanted interdigital transducers (IDTs) is used to generate surface acoustic waves (SAWs) to initiate droplet rotation. The inclined angle of the slanted interdigital transducers (IDTs) varies the spacing between the electrodes. These varied spaces allow for various working frequencies to be applied along the width of the slanted IDTs. The different frequencies can therefore generate a SAW at different positions on the piezoelectric substrate, which can correspond better to the droplet size. The two SAWs propagate along opposite directions, enter the droplet through two locations and drive the rotation of the droplet that is placed in a polydimethylsiloxane (PDMS) ring holder (Fig. 1a). The restriction from the PDMS ring holder induces a leveraging droplet resonance that increases the rotation rate and results in significant droplet deformation. These two effects induce microstreaming in the droplet more strongly than the classical oscillation dynamics of droplets^52,53^. The detailed mechanism of acoustofluidic-derived droplet rotation and simulation of microstreaming in the droplet are described in the supplementary material.

Synchronous drug loading and exosome encapsulation are achieved by acoustic radiation force, acoustic microstreaming, and shear stresses induced by the acoustofluidic drug loading device. The imbalanced body force acting on the side of the droplet causes rotation along with the generation of high-flow-rate vortex microstreaming within. During this process, acoustic radiation force and microstreaming-induced drag force comprehensively produce a trapping effect on particles in the middle of the droplet, such as drugs (e.g., doxorubicin), exosomes, and silica nanoparticles. While these particles are concentrated toward the center of the droplet, the frequency of particle–particle contact is significantly increased, resulting in higher drug loading efficiencies. Furthermore, the strong acoustic microstreaming in the droplet causes shear stress on the particles in the fluid field^54^. Since our previous research^50^ demonstrated that the rotating droplet induces a complex microstreaming environment that maintains a high-shear rate within the droplet, the exosome membrane could be disrupted by microstreaming-induced shear stress. This acoustic microstreaming-derived lipid membrane disruption causes an effect similar to a vortex or ultrasonic disruption and enhances the encapsulation of silica nanoparticles in the exosome membrane^55^. Since droplet rotation can result in both sample enrichment and membrane disruption effects, the acoustofluidic drug loading platform achieves drug loading and exosome encapsulation in one step (Fig. 1b).

To evaluate the concentration effect of the acoustofluidic drug loading device, we conducted both computational and experimental investigations to analyze the flow pattern and concentration effects. The microstreaming pattern in the rotating droplet shown in Fig. 1c demonstrates a vortex for trapping the particles in the center area of the droplet. The acoustic microstreaming-derived shear stress distribution is also shown in Fig. 1c. Although the high-shear-stress areas are distributed in the outer ring of the droplet and decrease in the center, the exosome fluorescence distribution (Fig. 1d) during droplet rotation indicates that exosomes are not entirely concentrated in the center of the droplets and can be exposed under high-shear stress. Furthermore, the drag and pressure gradient forces generated by acoustic microstreaming increase the drug/silica nanoparticle concentration and exosome membrane disruption. The concentrating effects of 50 nm porous silica nanoparticles and doxorubicin are also demonstrated in Fig. 1d. Compared with the uniform, low-intensity fluorescent distribution when the acoustofluidic device is inactivated, all groups show high-intensity fluorescence in the center of the droplet when the device is activated. Although the high-fluorescence areas differ among the 50 nm silica nanoparticles, exosomes, and doxorubicin, the varying fluorescent areas indicate that the device’s concentrating capabilities are dependent on the particles’ physical properties, such as size, shape, and density. The overlap of the varying concentration areas indicates that the acoustofluidic device can increase the effective drug concentration, drug loading efficiency, and drug loading rate and enable exosome encapsulation.

### Acoustofluidic-derived silica nanoparticle encapsulation

**Fig. 2:**
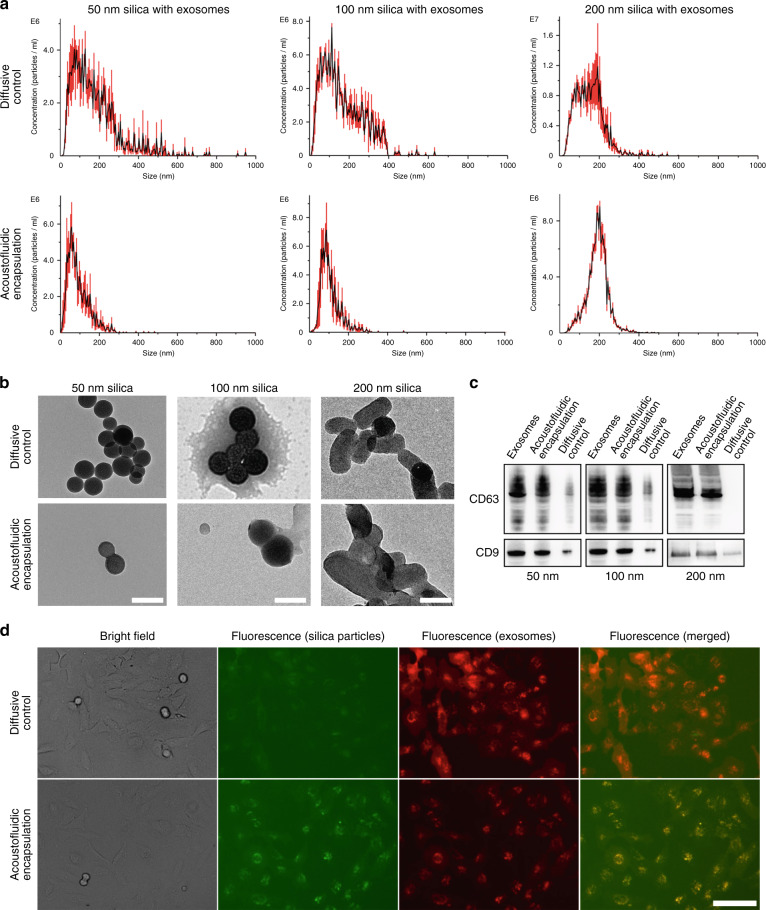
Acoustofluidics enhance the encapsulation of exosomes on the nanoparticles. **a** Size distributions for various mixtures of exosomes and silica nanoparticles. Compared with the diffusive control that only mixed exosomes and nanoparticles, acoustofluidic encapsulation exhibits narrower size distribution peaks and more closely resembles the size of the silica nanoparticles, indicating that the exosomes envelop the nanoparticles, error bar: SE, sample size: 5. **b** TEM images of silica particles of the diffusive control and after acoustofluidics encapsulation. In the diffusive control groups, the boundaries of the silica nanoparticles are clear. In contrast, the acoustofluidic encapsulation groups have blurred boundaries and show an additional layer, indicating exosome envelopment. **c** Western blot results show higher levels of exosomal surface biomarkers for the acoustofluidic encapsulation group than the diffusive control group. This observation indicates that acoustic treatment enhances silica nanoparticles with exosome membrane components. **d** HeLa endocytosis of 50 nm silica nanoparticles is enhanced by acoustofluidic encapsulation. Compared with the diffusive control group, which shows silica nanoparticles with lower levels of green fluorescence, the acoustofluidic encapsulation group shows higher green fluorescence levels overlapping with the exosomes’ red fluorescence. This observation indicates that the endocytosis of silica nanoparticles is enhanced when encapsulated within exosomes. Scale bar: **b** 100 nm, **d** 100 µm.

To validate whether the acoustofluidic-derived nanoparticle encapsulation device can be applied to nanoparticles with various shapes, we used 50 and 100 nm spherical and 200 nm rod silica nanoparticles for exosome encapsulation. To avoid the influence of concentration on the encapsulation process, the three different nanoparticles were kept at the same concentration of 400 µg/mL, and the exosome concentration was maintained at 1 × 10^7^ particles/mL during the encapsulation process. The particle size distributions of diffusive encapsulation groups and acoustofluidics-processed groups were evaluated by nanoparticle tracking analysis (NTA). As Fig. 2a shows, all diffusive control groups demonstrate size distribution results that contain multiple peaks within a large size range since these results contain size distribution curves from both the silica particles and remaining exosomes. The polydispersity of the exosome size distribution within the diffusive control groups widened the size distribution of the sample. However, the acoustofluidic encapsulation groups demonstrate clear peaks similar to the silica nanoparticle size distributions of 50, 100, and 200 nm, as shown in Fig. S1. By comparing the size distribution curves between the diffusive control groups and acoustofluidic encapsulation groups, it was found that the polydispersity of particle sizes significantly decreased in the acoustofluidic encapsulation groups. Since only exosomes and silica nanoparticles were added to each group and silica nanoparticles could be distinguished by their known sizes, particles showing polydisperse size distributions should exhibit free-floating exosomes, and thus, polydispersity should decrease after acoustofluidic encapsulation. Although the size of exosome-encapsulated silica nanoparticles was not significantly different from that of pure silica nanoparticles, the exosome envelope could be smaller than the NTA sensitivity limit. Transmission electron microscopy imaging (Fig. 2b) was further applied for morphology analysis of the encapsulated nanoparticles. Compared to the diffusive encapsulation control groups, nanoparticles processed by the acoustofluidic device demonstrated an outer membrane on the surface. This structure indicates that the exosome membrane successfully enveloped the silica nanoparticles. Since exosome membrane proteins cause endocytosis and can target cells, immunoblot technology was utilized to evaluate the existence of exosomal proteins on the nanoparticles. To confirm the presence of exosomal proteins on the encapsulated silica nanoparticles, samples from the diffusive controls and the acoustofluidic encapsulation groups were centrifuged at 10,000 × *g* in preparation for an immunoblot assay. The pellet was collected, and the supernatant was discarded. Since the exosomes could not be centrifuged at this speed, the centrifugation process eliminated the proteins from the free exosomes. The western blot results (Fig. 2c) show that the acoustofluidics-processed nanoparticles contained higher levels of exosome biomarkers CD9 and CD63 than nanoparticles diffused only in solution with the exosomes. These results demonstrate that the acoustofluidic device can encapsulate silica particles with exosomes and keep exosome-specific proteins intact. Thus, this acoustofluidic-based encapsulation process is independent of nanoparticle shape and size.

### Acoustofluidic-driven exosome encapsulation enhances endocytosis

Exosome membrane coating has been proven to promote the cellular uptake of nanoparticles. To validate whether acoustofluidic-derived encapsulation can have similar effects, green fluorescent-tagged silica nanoparticles and red fluorescent-tagged exosomes were processed by an acoustofluidic device and used to culture HeLa cells. Compared with that in the diffusive encapsulation samples, the green fluorescence of silica nanoparticles was significantly stronger in the acoustofluidic encapsulated samples (Fig. 2d). This finding means that acoustofluidics-processed exosome encapsulation increases the intracellular intake of silica nanoparticles. Furthermore, in the acoustofluidic encapsulated samples, the exosome and nanoparticle fluorescence displays high spatial overlap, showing that exosomes and nanoparticles are delivered as integrated particles. Acoustofluidic-derived encapsulation promotes the endocytosis of silica nanoparticles with different shapes and sizes (Fig. S2), suggesting tremendous versatility in terms of which nanoparticles can be utilized.

### Acoustofluidic rotation increases the drug loading efficiency and drug loading rate

**Fig. 3:**
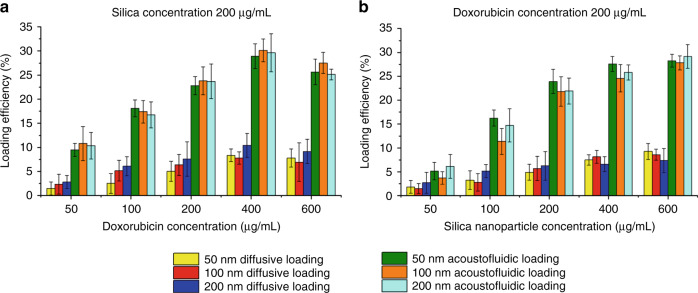
An acidic fluorescent drug loading device enhances the doxorubicin loading efficiency with porous silica nanoparticles. **a** The doxorubicin loading efficiency for 50, 100, and 200 nm silica nanoparticles. The difference between free loading and acoustofluidic loading with a constant nanoparticle concentration of 200 μg/mL and increasing doxorubicin concentration is shown. **b** The doxorubicin loading efficiency for 50, 100, and 200 nm silica nanoparticles. The difference between free loading and acoustofluidic loading with a constant doxorubicin concentration of 200 μg/mL and increasing nanoparticle concentration is shown, error bar: SE, replicates: 3.

Since both the drug loading efficiency and drug loading rate of the porous silica particles depend on the concentration of the drug and nanoparticles, the doxorubicin and silica particle concentration were both varied, while the other variables were held constant. The doxorubicin concentration was analyzed by absorbance of doxorubicin solution at 480 nm; the loading efficiency was evaluated by the ratio between the concentration of free doxorubicin before and after acoustofluidic drug loading. The doxorubicin loading efficiency of 50, 100, and 200 nm porous silica nanoparticles is demonstrated in Fig. 3. The acoustofluidics-processed samples show higher loading efficiencies than the control groups, as presented in Fig. 3a. The exosome concentration was maintained at 1 × 10^7^ particles/mL for all experiments. We also observed that the loading efficiency decreased after the doxorubicin concentration reached 400 µg/mL. This result is due to the saturation of silica and exosome particles’ drug loading capabilities. The comparison of doxorubicin concentration among the groups shows that the acoustofluidic drug loading device can achieve a higher loading efficiency than the control groups (Fig. 3b). Additionally, when compared with the control groups processed by 30 min of diffusive loading, the groups with 2 min of acoustofluidic drug loading show both a higher loading efficiency and faster loading rate. This finding effectively reemphasizes the idea that acoustofluidics-derived drug loading has the potential to load a wide range of nanoparticles of various shapes and sizes more efficiently and rapidly than current methods.

### Drug intake improvement and proliferation inhibition via the acoustofluidic drug loading device

**Fig. 4:**
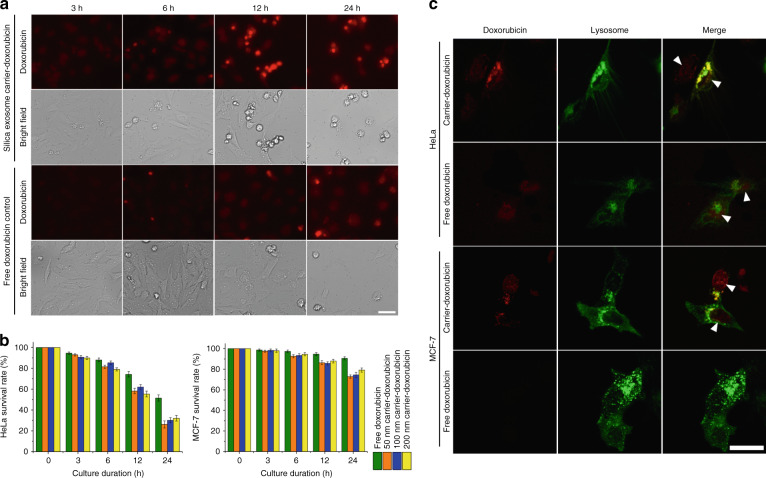
Enhanced doxorubicin drug intake efficiency with acoustofluidic drug loading-derived nanoparticles. **a** Microscope images of HeLa cells intaking doxorubicin-loaded 50 nm silica exosome carriers. The images show higher intracellular doxorubicin fluorescence than that of free doxorubicin. Scale bar: 50 µm. **b** The MTT viability assay indicates that the presence of doxorubicin delivered by silica exosome carriers decreases the viability of HeLa and MCF-7 cells, error bar: SE, replicates: 3. **c** The doxorubicin in silica exosome nanocarriers is delivered into cells by endocytosis, as exhibited by the overlapping red (doxorubicin) and green (lysosome staining) fluorescence in both HeLa and MCF-7 cells. The white triangles point to the doxorubicin within the nucleic areas of cells. Free and carrier doxorubicin-treated HeLa cells and carrier doxorubicin-treated MCF-7 cells all demonstrate doxorubicin fluorescence in the nucleic areas. Scale bar: 30 µm.

To evaluate the technology’s potential in drug delivery to tumor cells, we used the acoustofluidic device to process doxorubicin, exosomes, and 50, 100, and 200 nm porous silica nanoparticles, and the products were used for culture with HeLa and MCF-7 tumor cells. The survival rates of cells, which were evaluated by MTT cell viability assays, were used for treatment efficiency analysis. The control groups were treated with free doxorubicin, and the final doxorubicin concentration in the culture medium was 2.5 µg/mL for both free doxorubicin and doxorubicin-loaded by the acoustofluidic device. During 24 h of culture, fluorescence microscopy images demonstrated that acoustofluidic-loaded doxorubicin in 50 nm porous silica nanoparticles exhibited stronger intracellular doxorubicin fluorescence and lower cell density (Fig. 4a and Fig. S3). All doxorubicin-loaded groups showed lower cell viability than the free doxorubicin groups in both HeLa and MCF-7 cells (Fig. 4b). To further investigate the exosome-silica-doxorubicin uptake mechanism, we utilized a GFP-tagged lysosome tracker prestaining of the cells. Since doxorubicin is a small molecule belonging to the anthracycline family, the uptake of doxorubicin by cells can be achieved by diffusion^56^. However, multiple cell lines have shown resistance to doxorubicin by the overexpression of P-glycoprotein, which is a transporter on the cell membrane that can actively transport doxorubicin out of cells^57^. For the cell lines in this research, HeLa cells have shown low resistance to doxorubicin because of the low expression of P-glycoprotein^58^, while MCF-7 has been commonly used as a doxorubicin-resistant cell line in the scientific literature^57–59^. Although free doxorubicin can be distributed into both cell lines and stains the cell nuclei in an overlapping pattern as indicated by the fluorescence results (Fig. 4c), the doxorubicin fluorescence strength in the nucleic areas of MCF-7 cells is significantly weaker than that in the nucleic areas of HeLa cells. This decrease in fluorescence strength indicates the active pumping of doxorubicin by P-glycoprotein, thereby causing a lower abundance and shorter existence period of doxorubicin in resistant cell lines. Although the acoustofluidics-constructed drug carriers did not efficiently kill the MCF-7 cells, the doxorubicin delivered by carriers achieves twice the death rate of free doxorubicin, indicating the acoustofluidics-constructed carriers’ promotion of therapeutic efficiency. Furthermore, cells cultured with exosome-silica-doxorubicin, as presented in Fig. 4c, show a different doxorubicin distribution pattern. Doxorubicin fluorescence in these groups was distributed in both the nucleic areas and lysosome areas, as demonstrated by the overlapping fluorescence from the lysosome-specific staining dye. Since the formation of lysosomes involves the endocytosis pathway, a common exosome intake process^13^, doxorubicin loaded in exosome-silica is transported by cells through exosome intake mechanisms. Since the doxorubicin delivered by exosome-silica particles first exists in the lysosome, drug molecules cannot be pumped out rapidly by P-glycoprotein and can reside longer inside the cells and cause higher abundance in nucleic areas, as demonstrated by the higher doxorubicin fluorescence in MCF-7 nucleic areas than in MCF-7 cells cultured with free doxorubicin. The confocal images in Fig. 4c also show red fluorescence from doxorubicin in the nucleic areas of free and carrier doxorubicin-treated HeLa cells and carrier doxorubicin-treated MCF-7 cells, indicating that doxorubicin delivered by acoustofluidics-constructed carriers can still diffuse to functioning areas. This difference in the intake mechanism can overcome many limitations of drug-resistant cell lines.

## Discussion

In summary, we developed an acoustofluidic device that simultaneously achieved drug loading and exosome encapsulation. Although the streaming within exosomes is not considered in this article, as it has been for other unilamellar vesicles^60^, it would be of value to consider that analysis for future works. The acoustofluidic device generates three effects on particles within a droplet: acoustic radiation force, associated vortex microstreaming, and microstreaming-derived shear stress. These effects induce the drug molecules, exosomes, and inert nanocarriers to concentrate at the droplet center. Once the particles are concentrated within a smaller volume, the higher interaction probabilities promote increased drug loading efficiency. The drug loading efficiencies and loading rates associated with this acoustofluidic device are higher than those measured using vortex-diffusive loading for any concentration of doxorubicin and silica. A drug loading efficiency of 30% was achieved, which is significantly higher than those of inert material nanocarriers with exosome encapsulation by passive incubation. Acoustofluidic drug loading also shows an 11.68% higher loading rate than that achieved by sonication, which was used for exosome drug loading as an active approach^61^. Although the low volume of the droplet limits the throughput of the device, this limitation could potentially be solved by driving multiple droplet rotations synchronously by a similar pair of IDTs. Apart from the high loading efficiency due to increased concentration effects, the induced acoustic microstreaming within the droplet increased the local shear stress. This effect disrupts the membrane of the exosomes and promotes exosome membrane encapsulation of the silica particles. A membrane-like structure was found, and exosomal surface protein biomarkers were identified in the nanoparticles. The biomarkers indicate that the nanoparticles have acquired an exosomal membrane and have the potential for targeted intracellular transport. In vitro cell intake experiments confirmed that acoustofluidic-derived exosome encapsulation significantly promotes the endocytosis of nanoparticles. This acoustofluidic-derived drug carrier also shows potential for resolving problems with drug-resistant cell lines. Since the drug delivered by the exosome-silica nanocarriers is transported via endocytosis pathways, it will be released from the endocytosis-formed lysosome and further released into the cell. This intake mechanism avoids the rapid elimination of the drug by actively transporting proteins on the cell membrane and sustains a high concentration of drug inside cells, which can significantly improve the treatment outcome for cancer derived from drug-resistant cells. Combined with exosome encapsulation, silica nanoparticles loaded with doxorubicin have higher drug intake efficiencies, which subsequently inhibits tumor cell proliferation.

The many advantages of this acoustofluidic drug loading system make it feasible to serve as a nanocarrier-based drug delivery manufacturing system. The mechanisms for particle enrichment and the enhanced local shear stresses arising from acoustic microstreaming are both physical effects and work independently of chemical composition. Although the contribution of acoustic radiation force, acoustic streaming, and microstreaming-derived shear stress on drug loading and vesicle encapsulation still need to be further evaluated, the phenomenon that we found offers an alternative approach for drug loading. Compared with conventional physical methods such as passive diffusion and sonication, which can take hours to complete, the acoustofluidic device requires only a few minutes. Furthermore, this device shows tolerance to differences in the morphological properties of nanoparticles by enabling drug loading and exosome encapsulation of nanoparticles with different shapes and sizes. Acoustofluidic drug loading technology provides a unique physical process for improving the drug loading efficiency of inert nanocarriers. As a result, this technology’s adaptability allows for applications with a variety of drug types. Furthermore, acoustofluidic drug loading technology facilitates exosome encapsulation during drug loading, enabling the recruitment of exosomes. Since exosomes are involved in intercellular communication, the introduction of exosome encapsulation can potentially improve targeted delivery and intake. The ability of acoustofluidic drug loading technology to physically generate hybrid drug carriers combines the advantages of varying drug delivery platforms, such as programmable release and targeted delivery from nanoparticle-exosome-based drug carriers^3,62^, deriving innovative and powerful drug carriers.

## Materials and methods

### Device fabrication

To fabricate the acoustofluidic drug loading device, one pair of IDTs was designed by AutoCAD (Autodesk, USA), and a Y+128° X-propagation lithium niobate (LiNbO_3_) substrate was used for IDT deposition. The deposition was achieved by photolithography procedures. In particular, the substrate and the mask were aligned by an MA/BA6 mask aligner (SUSS MicroTec., Germany), and the SPR3012 photoresist (MicroChem Corp., USA) was exposed to UV light. After applying CD26 developing solution (MicroChem Corp., USA) to remove unwanted photoresist, an e-beam evaporator (Semicore Corp., USA) deposited a metal double layer (Cr/Au, 50 Å/500 Å) on the substrate. This step was followed by a lift-off process through a PRS3000 resist stripper (VWR, USA), forming the electrodes. The height of the IDT electrodes was 50 nm, and the finger spacing of the IDT ranged from 75 to 35 μm. A 2.2 mm diameter and 0.55 mm height PDMS ring was prepared by Curing Agent and Base of Silicone Elastomer (Dow Corning, USA) and bonded on the substrate to hold the droplet.

### Silica nanoparticle synthesis

To synthesize 50 and 100 nm porous silica spherical particles and 200 nm porous silica rods, 0.11, 0.21, and 0.36 g/mL cetyltrimethylammonium bromide (CTAB, Sigma–Aldrich, USA) water suspensions were prepared by 15 min sonication in 35 °C incubation, respectively; then, 0.75, 0.9, and 1.2 mL of NH_3_H_2_O (Sigma–Aldrich, USA) were added to each CTAB suspension, followed by 10 min of vortexing. Tetraethyl orthosilicate (TEOS, Sigma–Aldrich, USA) was then added dropwise with total volumes of 0.45, 0.6, and 0.72 mL. The silica nanoparticles tagged with green fluorescence were created by adding 45, 60, and 72 µL of fluorescein isothiocyanate-conjugated (3-aminopropyl)triethoxysilane (APS, Sigma–Aldrich, USA) solution (1/19, mol/mol). After mixing at room temperature for 3 h, the synthesized nanoparticles were collected by centrifugation at 13,000 × *g*, followed by washing with ethanol two times. The porous silica nanoparticles were then collected by centrifugation and washed with sterilized distilled water three times for further use.

### Acoustofluidic encapsulation and drug loading

The acoustofluidic drug loading device was driven by a function generator (Agilent, USA) and an amplifier (Amplifier Research, USA). The substrate was attached to a Peltier cooling system (Hebei IT, China) powered by a variable DC power supply (Tekpower, USA). Droplet rotation was monitored with an upright microscope (Olympus, Japan) combined with a CCD camera (Photometrics, USA). For exosome encapsulation analysis, the 10 µL droplet contained 1 × 10^7^ particles/mL human plasma exosomes (BioVision, USA) and 400 µg/mL porous silica nanoparticles. Diffusive encapsulation control was derived by 10 sec of vortexing for initial mixing and incubated for 30 min. The products were analyzed by a nanoparticle tracking analysis (Malvern, England) system to evaluate the size of the encapsulated nanoparticles. Drug loading efficiency analysis used 10 µL droplets containing 400 µg/mL silica nanoparticles and 50, 100, 200, 400, and 600 µg/mL doxorubicin or containing 400 µg/mL doxorubicin and 50, 100, 200, 400, and 600 µg/mL silica nanoparticles. Doxorubicin hydrochloride (Sigma–Aldrich, USA) was dissolved in water and PBS solution as a 1 mg/mL stock solution and further diluted in water to varying concentrations used in the experiment. The pH of the water and PBS solution was measured to be 7.1. The diffusive loading control was derived by 2 min of vortexing and 30 min of diffusive loading. The doxorubicin concentration was analyzed by the solution’s absorption at 480 nm by a microplate reader (BioTek, USA). The synchronous exosome encapsulation and drug loading experiment used a 10 µL droplet containing 1 × 10^7^ particles/mL human plasma exosomes, 400 µg/mL silica nanoparticles, and 400 µg/mL doxorubicin. All encapsulation and drug loading procedures were achieved using a 30 V input voltage with a 23 MHz frequency. The entire process lasted 2 min.

### Transmission electron microscopy

Ten microliters of exosome encapsulation product was covered by a support film of a 300-mesh copper grid (Electron Microscopy Sciences, USA) for 20 min of absorption. After 3 min of washing with water three times, uranyl–acetate solution was used to incubate the grids for 10 min. The grids were then washed with water three times to complete negative staining. Finally, an electron microscope (FEI Company, USA) was used to image the grids.

### Immunoblot

To avoid interference from free exosomes not attached to nanoparticles, 10,000 × *g* centrifugation was applied to separate nanoparticles. Isolated nanoparticles were then resuspended and analyzed by immunoblotting. Mouse anti-CD9 (ab13492, 1 μg/mL, Abcam, USA) and mouse anti-CD63 (Santa Cruz, USA) were used as the primary antibodies. Goat anti-mouse IgG (ab97040, 0.05 μg/mL, Abcam, USA) was used as a secondary antibody. Protein abundance was analyzed with a ChemiDoc XRS+ system (Bio-Rad, USA).

### Cell culture and microscopy

HeLa cells (ATCC, USA) and MCF-7 cells (ATCC, USA) were grown in DMEM (Gibco, Life Technologies, MA, USA) with culture media containing 10% fetal bovine serum (Gibco, USA) and penicillin–streptomycin (Gibco, USA). Cells were cultured in a Nu-4750 incubator (NuAire, USA) at 37 °C with a CO_2_ level of 5%. The concentrations of silica nanoparticles or exosome-encapsulated silica nanoparticles for HeLa cell intake experiments were 10 µg/mL in a culture medium for 6 h. After changing the culture medium to discard free nanoparticles, a fluorescent cell imager (Bio-Rad, USA) was used, capturing the silica distribution within the cell. For drug delivery experiments, exosome-encapsulated silica nanoparticles loaded with doxorubicin and free doxorubicin were added to the culture medium to a final doxorubicin concentration of 2.5 µg/mL. MTT cell viability assays (Thermo Fisher, USA) and fluorescence microscopy were used to analyze the viability of HeLa and MCF-7 cells and intracellular doxorubicin fluorescence 3, 6, 12, and 24 h after culturing. A confocal microscope (Zeiss, Germany) was used to record the doxorubicin and silica nanoparticle fluorescence distribution 6 h after culture, including lysosome staining by LysoTracker Green (Thermo Fisher, USA) during this period.

## Supplementary information

SI revision

## References

[1] Hui Y (2019). Role of nanoparticle mechanical properties in cancer drug delivery. ACS nano.

[2] Phan DT (2017). A vascularized and perfused organ-on-a-chip platform for large-scale drug screening applications. Lab Chip.

[3] Blanco E, Shen H, Ferrari M (2015). Principles of nanoparticle design for overcoming biological barriers to drug delivery. Nat. Biotechnol..

[4] Singh R, Lillard JW (2009). Nanoparticle-based targeted drug delivery. Exp. Mol. Pathol..

[5] Cai SS, Li T, Akinade T, Zhu Y, Leong KW (2021). Drug delivery carriers with therapeutic functions. Adv. Drug Deliv. Rev.

[6] Kovalainen M (2013). Development of porous silicon nanocarriers for parenteral peptide delivery. Mol. Pharm..

[7] Wang S, Sun J, Zhang D, Wong P (2016). A nanobiosensor for dynamic single cell analysis during microvascular self-organization. Nanoscale.

[8] Shen S, Wu Y, Liu Y, Wu D (2017). High drug-loading nanomedicines: progress, current status, and prospects. Int. J. Nanomed..

[9] Kooijmans SA, Vader P, van Dommelen SM, van Solinge WW, Schiffelers RM (2012). Exosome mimetics: a novel class of drug delivery systems. Int. J. Nanomed..

[10] Nawijn C (2021). Multi-timescale microscopy methods for the characterization of fluorescently-labeled microbubbles for ultrasound-triggered drug release. J. Vis. Exp.

[11] Smyth T (2015). Biodistribution and delivery efficiency of unmodified tumor-derived exosomes. J. Control. Release.

[12] Milane L, Singh A, Mattheolabakis G, Suresh M, Amiji MM (2015). Exosome mediated communication within the tumor microenvironment. J. Control. Release.

[13] Hessvik NP, Llorente A (2018). Current knowledge on exosome biogenesis and release. Cell. Mol. Life Sci..

[14] Bobrie A, Colombo M, Raposo G, Théry C (2011). Exosome secretion: molecular mechanisms and roles in immune responses. Traffic.

[15] Yong T (2019). Tumor exosome-based nanoparticles are efficient drug carriers for chemotherapy. Nat. Commun..

[16] Tanziela T, Shaikh S, Jiang H, Lu Z, Wang X (2020). Efficient encapsulation of biocompatible nanoparticles in exosomes for cancer theranostics. Nano Today.

[17] Cheng G (2018). Self-assembly of extracellular vesicle-like metal–organic framework nanoparticles for protection and intracellular delivery of biofunctional proteins. J. Am. Chem. Soc..

[18] Wang Z (2020). Acoustofluidic salivary exosome isolation: a liquid biopsy compatible approach for human papillomavirus–associated oropharyngeal cancer detection. J. Mol. Diagn..

[19] Wu M (2019). Acoustofluidic separation of cells and particles. Microsyst. Nanoeng..

[20] Dai Nguyen T (2020). Acoustofluidic closed-loop control of microparticles and cells using standing surface acoustic waves. Sens. Actuators B: Chem..

[21] Xie Y (2020). Acoustic cell separation based on density and mechanical properties. J. Biomech. Eng..

[22] Zhang P, Bachman H, Ozcelik A, Huang TJ (2020). Acoustic microfluidics. Annu. Rev. Anal. Chem..

[23] Zhang SP (2018). Digital acoustofluidics enables contactless and programmable liquid handling. Nat. Commun..

[24] Guo F (2015). Controlling cell–cell interactions using surface acoustic waves. Proc. Natl Acad. Sci. USA.

[25] Cox L, Melde K, Croxford A, Fischer P, Drinkwater BW (2019). Acoustic hologram enhanced phased arrays for ultrasonic particle manipulation. Phys. Rev. Appl..

[26] Ray A (2020). Holographic detection of nanoparticles using acoustically actuated nanolenses. Nat. Commun..

[27] Läubli NF (2021). 3D mechanical characterization of single cells and small organisms using acoustic manipulation and force microscopy. Nat. Commun..

[28] Wang W (2016). Enhanced sensitivity of temperature-compensated SAW-based current sensor using the magnetostrictive effect. Smart Mater. Struct..

[29] Liu Z (2021). Theory of acoustophoresis in counterpropagating surface acoustic wave fields for particle separation. Phys. Rev. E.

[30] Tian Z (2020). Dispersion tuning and route reconfiguration of acoustic waves in valley topological phononic crystals. Nat. Commun..

[31] Tian Z (2019). Wave number–spiral acoustic tweezers for dynamic and reconfigurable manipulation of particles and cells. Sci. Adv..

[32] Gong Z, Baudoin M (2021). Three-dimensional trapping and dynamic axial manipulation with frequency-tuned spiraling acoustical tweezers: a theoretical study. Phys. Rev. Appl..

[33] Koo K-i, Lenshof A, Huong LT, Laurell T (2021). Acoustic cell patterning in hydrogel for three-dimensional cell network formation. Micromachines.

[34] Olofsson K, Hammarström B, Wiklund M (2020). Acoustic separation of living and dead cells using high density medium. Lab Chip.

[35] Mutafopulos K (2019). Traveling surface acoustic wave (TSAW) microfluidic fluorescence activated cell sorter (μFACS). Lab Chip.

[36] Ley MW, Bruus H (2017). Three-dimensional numerical modeling of acoustic trapping in glass capillaries. Phys. Rev. Appl..

[37] Zhu H (2021). Acoustohydrodynamic tweezers via spatial arrangement of streaming vortices. Sci. Adv..

[38] Wang W (2014). Acoustic propulsion of nanorod motors inside living cells. Angew. Chem. Int. Ed..

[39] Tayebi M, Zhou Y, Tripathi P, Chandramohanadas R, Ai Y (2020). Exosome purification and analysis using a facile microfluidic hydrodynamic trapping device. Anal. Chem..

[40] Barad H-N, Kwon H, Alarcón-Correa M, Fischer P (2021). Large area patterning of nanoparticles and nanostructures: current status and future prospects. ACS nano.

[41] Kim M, Bayly PV, Meacham JM (2021). Motile cells as probes for characterizing acoustofluidic devices. Lab Chip.

[42] Alhasan L (2016). Rapid enhancement of cellular spheroid assembly by acoustically driven microcentrifugation. ACS Biomater. Sci. Eng..

[43] Yeo LY, Friend JR, McIntosh MP, Meeusen EN, Morton DA (2010). Ultrasonic nebulization platforms for pulmonary drug delivery. Expert Opin. drug Deliv..

[44] Collins DJ, Neild A, DeMello A, Liu A-Q, Ai Y (2015). The Poisson distribution and beyond: methods for microfluidic droplet production and single cell encapsulation. Lab Chip.

[45] Destgeer G (2019). Microparticle self-assembly induced by travelling surface acoustic waves. RSC Adv..

[46] Belling JN (2020). Acoustofluidic sonoporation for gene delivery to human hematopoietic stem and progenitor cells. Proc. Natl Acad. Sci. USA.

[47] Zhao S (2020). A disposable acoustofluidic chip for nano/microparticle separation using unidirectional acoustic transducers. Lab Chip.

[48] Li H, Friend JR, Yeo LY (2007). Surface acoustic wave concentration of particle and bioparticle suspensions. Biomed. microdevices.

[49] Rogers PR, Friend JR, Yeo LY (2010). Exploitation of surface acoustic waves to drive size-dependent microparticle concentration within a droplet. Lab Chip.

[50] Gu Y (2021). Acoustofluidic centrifuge for nanoparticle enrichment and separation. Sci. Adv..

[51] Ding C, Li Z (2017). A review of drug release mechanisms from nanocarrier systems. Mater. Sci. Eng.:.

[52] Ashgriz, N. *Handbook of Atomization and Sprays: Theory and Applications* (Springer Science & Business Media, 2011).

[53] Hill R, Eaves L (2008). Nonaxisymmetric shapes of a magnetically levitated and spinning water droplet. Phys. Rev. Lett..

[54] Wang Z (2019). Cell lysis via acoustically oscillating sharp edges. Lab Chip.

[55] Shin S, Ault JT, Stone HA (2015). Flow-driven rapid vesicle fusion via vortex trapping. Langmuir.

[56] Arora HC (2012). Nanocarriers enhance doxorubicin uptake in drug-resistant ovarian cancer cells. Cancer Res..

[57] Meng H (2010). Engineered design of mesoporous silica nanoparticles to deliver doxorubicin and P-glycoprotein siRNA to overcome drug resistance in a cancer cell line. ACS nano.

[58] Li J-M (2012). Multifunctional QD-based co-delivery of siRNA and doxorubicin to HeLa cells for reversal of multidrug resistance and real-time tracking. Biomaterials.

[59] Zampieri L, Bianchi P, Ruff P, Arbuthnot P (2002). Differential modulation by estradiol of P-glycoprotein drug resistance protein expression in cultured MCF7 and T47D breast cancer cells. Anticancer Res..

[60] Pereno V, Lei J, Carugo D, Stride E (2020). Microstreaming inside model cells induced by ultrasound and microbubbles. Langmuir.

[61] Fu S, Wang Y, Xia X, Zheng JC (2020). Exosome engineering: current progress in cargo loading and targeted delivery. NanoImpact.

[62] Antimisiaris SG, Mourtas S, Marazioti A (2018). Exosomes and exosome-inspired vesicles for targeted drug delivery. Pharmaceutics.

