# Supplementary material for: Acoustofluidics for simultaneous nanoparticle-based drug loading and exosome encapsulation
Source: Microsyst Nanoeng. 2022 Apr 28;8:45. doi: 10.1038/s41378-022-00374-2 (PMC9051122; doi:10.1038/s41378-022-00374-2)
Supplement: Supplementary file 1 — SI revision [file 41378_2022_374_MOESM1_ESM.pdf]

Supplemental Information

Acoustofluidics for Simultaneous Nanoparticle-based Drug Loading and Exosome Encapsulation

Zeyu Wang[1], Joseph Rich[1], Nanjing Hao[1], Yuyang Gu[1], Chuyi Chen[1], Shujie Yang[1], Peiran Zhang[1], Tony Jun Huang[1*]

[1] Department of Mechanical Engineering and Materials Science, Duke University, NC 27708, USA

[*] To whom correspondence should be addressed. Email: tony.huang@duke.edu

Table of Contents:

1. Computation model
2. Size distributions of silica nanoparticles
3. Exosome encapsulation enhances endocytosis of multiple size nanoparticles.
4. Microscope images of doxorubicin-loaded, 50 nm silica nanoparticles encapsulated within exosomes being intaken by MCF-7 cells.
5. Acoustofluidics derived exosome encapsulation with drug loading showing an enhanced doxorubicin intake efficiency and inhibited cell proliferation.
6. Acoustofluidics derived exosome encapsulation with drug loading shows an enhanced doxorubicin intake efficiency and inhibited cell proliferation.

1. Mechanism and computational model

The classical oscillation of droplets can be described by equation S1:[1,2]

$$\omega_n^2 = n(n+1)(n-1)(n+2)\frac{\sigma}{r^3[(n+1)\rho_{liquid}+n\rho_{air}]} \quad \text{(S1)}$$

where n, r, σ, ρ_{liquid}, ρ_{air} are the spherical harmonic degree, droplet radius, surface tension, and the density of the liquid and air. In our acoustofluidic droplet rotation device the radius of the droplet is restricted by the PDMS ring (which's radius is a), so the relationship of the droplet's height (h), radius (r) and volume (V) could be described by equation S2:[3]

$$V - \pi a^2 h = \frac{\pi}{3}\left(r+\sqrt{r^2-a^2}\right)^2\left[3r-\left(r+\sqrt{r^2-a^2}\right)\right] \quad \text{(S2)}$$

Based on equation S2, radial velocity of the droplet could be described as equation S3:[3]

$$V_{drop-radial}(\varphi,t) = \frac{\partial r(\varphi,t)}{\partial t} = a[1-\varepsilon_0\sin(\omega_l t)\cos(l\varphi+\varphi_0)] \quad \text{(S3)}$$

Due to the droplet's closed volume, the propagation of the acoustic wave derives bulk acoustic streaming.[4] Since acoustic streaming is a time-average steady flow derived by the Reynold stress, which associates the gradient of the momentum flux, acoustic streaming forces fluids to flow with the acoustic energy flux dissipation. Therefore, vortex streaming could be generated in the droplet. By combining the droplet rotation described in equation S3 and the vortex streaming field, the particle motion trajectory can be described by equation S4:[3]

$$x_{radial} = \int_0^t V_{drop-radial} + V_{vortex-radial}\,dt \quad \text{(S4)}$$

Acoustic streaming in the droplet is governed by equations:[5,6]

$$\rho_0\nabla\cdot v = 0 \quad \text{(S5)}$$

$$\rho_0(v\cdot\nabla)v = -\nabla p + \mu\nabla^2 v + \left(\mu_b+\frac{1}{3}\mu\right)\nabla(\nabla\cdot v) + F \quad \text{(S6)}$$

where v, p, ρ_0, μ, and μ_b are the streaming velocity, pressure in the liquid, liquid density, shear viscosity, and the bulk viscosity. The leaky SAW propagation, which is relative to the acoustic particle velocity (v_1), activates acoustic streaming by the body force F:[5,6]

$$F_x = -\rho_0\left(\frac{\partial\overline{u_1u_1}}{\partial x}+\frac{\partial\overline{v_1u_1}}{\partial y}+\frac{\partial\overline{w_1u_1}}{\partial z}\right) \quad \text{(S7)}$$

$$F_y = -\rho_0\left(\frac{\partial u_1v_1}{\partial x}+\frac{\partial v_1v_1}{\partial y}+\frac{\partial w_1v_1}{\partial z}\right) \quad \text{(S8)}$$

$$F_z = -\rho_0\left(\frac{\partial\overline{u_1w_1}}{\partial x}+\frac{\partial\overline{v_1w_1}}{\partial y}+\frac{\partial\overline{w_1w_1}}{\partial z}\right) \quad \text{(S9)}$$

The leaky SAW velocity is described by equations:[7]

$$u_1 = 0 \quad \text{(S10)}$$

$$v_1 = i\omega\left(A_m e^{i\omega t}e^{-ik_L y}e^{-\alpha k_L z}\right) \quad \text{(S11)}$$

$$w_1 = i\omega\left(-i\alpha A_m e^{i\omega t}e^{-ik_L y}e^{-\alpha k_L z}\right) \quad \text{(S12)}$$

where A_m, k_L, and α are the substrate vibration amplitude, leaky SAW wave number, and attenuation coefficient, respectively.

By integrating equations S7-12, the streaming force could be described by: [3]

$$F_x = 0 \quad \text{(S13)}$$

$$F_y = -(1+\alpha_1^2)A_m^2\omega^2 k_i e^{[2(k_i y+\alpha_1 k_i z)]} \quad \text{(S14)}$$

$$F_z = -(1+\alpha_1^2)A_m^2\omega^2 k_i\alpha_1 e^{[2(k_i y+\alpha_1 k_i z)]} \quad \text{(S15)}$$

where $\alpha = i\alpha_1$.

From our previous work, the highest microstreaming velocity in the droplet achieved is 100 mm/s, which was investigated experimentally and numerically.[3] The microstreaming velocity during droplet rotation can achieve 100 mm/s and the generated shear stress could be calculated by equation S16.[8,9]

$$|\boldsymbol{\tau}| = \sqrt{\left(\boldsymbol{\mu}\frac{\partial \boldsymbol{u}}{\partial \boldsymbol{y}}\right)^{2} + \left(\boldsymbol{\mu}\frac{\partial \boldsymbol{v}}{\partial \boldsymbol{x}}\right)^{2}} \qquad \text{(S16)}$$

where μ is the fluid dynamic viscosity, and u and v are the fluid velocity of the x- and y-components, respectively.

The 3D numerical models that describe the flow field and particle motion on the horizontal section of the rotating droplet were solved by COMSOL Multiphysics 5.6.[10] A quasi-ellipse with radius of *R* was settled as the simulation domain. The flow field simulation is predefined as *laminar flow* governed by equation S5 and S6. The streaming force is governed by equation S14 and settled as the *volume force*. The model of the rotating droplet was derived by a *rotating domain* condition. By solving the *laminar flow* and the *rotating domain* interfaces together, the rotation of the droplet and the flow field induced by the propagation of the leaky-SAWs could be simulated.

2. Size distributions of silica nanoparticles

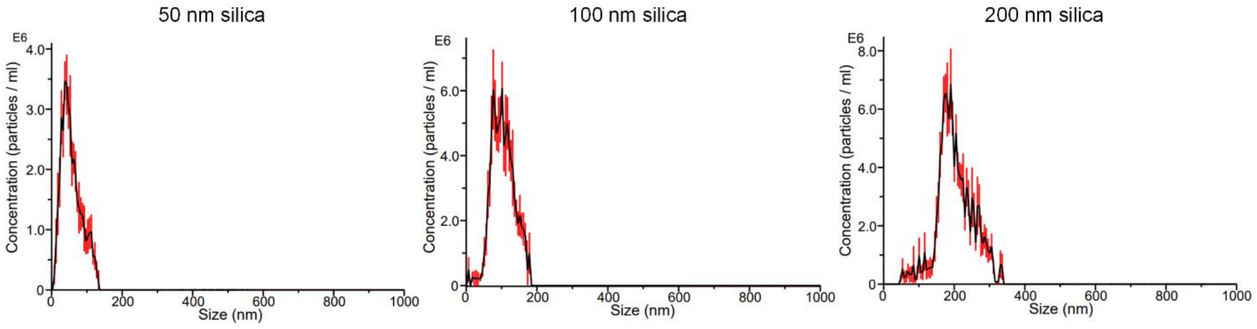

Figure S1. Size distribution of 50 nm, 100 nm, and 200 nm silica nanoparticles. Peaks of the highest concentrations in each sample are approximately the estimated sizes of the nanoparticles. error bar: SE, sample size: 5.

3. Exosome encapsulation enhances endocytosis of multiple size nanoparticles

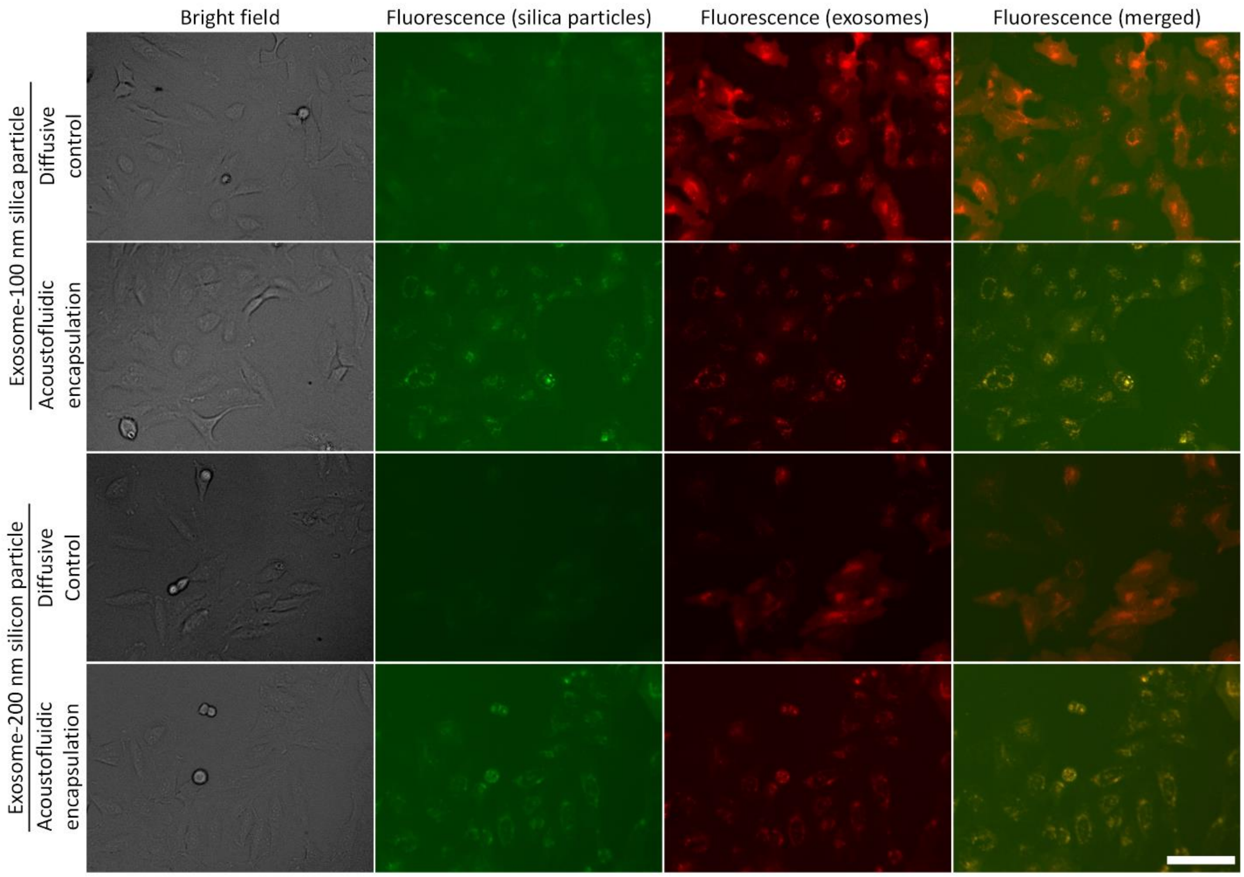

Figure S2. Exosome encapsulation enhances endocytosis of multiple size nanoparticles. For both 100 nm and 200 nm silica nanoparticles, nanoparticles processed by acoustofluidic encapsulation induce stronger nanoparticle fluorescence (green fluorescence) inside the cells compared to nanoparticles only processed by diffusive encapsulation controls. The acoustofluidic encapsulated nanoparticles' fluorescence shows overlap with exosomes' fluorescence (red fluorescence), indicating they are intaken by cells simultaneously. These results indicate endocytosis of 100 nm spheric and 200 nm rod silica nanoparticles by HeLa cells is enhanced after acoustofluidics derived exosome encapsulation. Scale bar: 100 µm.

4. Microscope images of doxorubicin-loaded, 50 nm silica nanoparticles encapsulated within exosomes being intaken by MCF-7 cells.

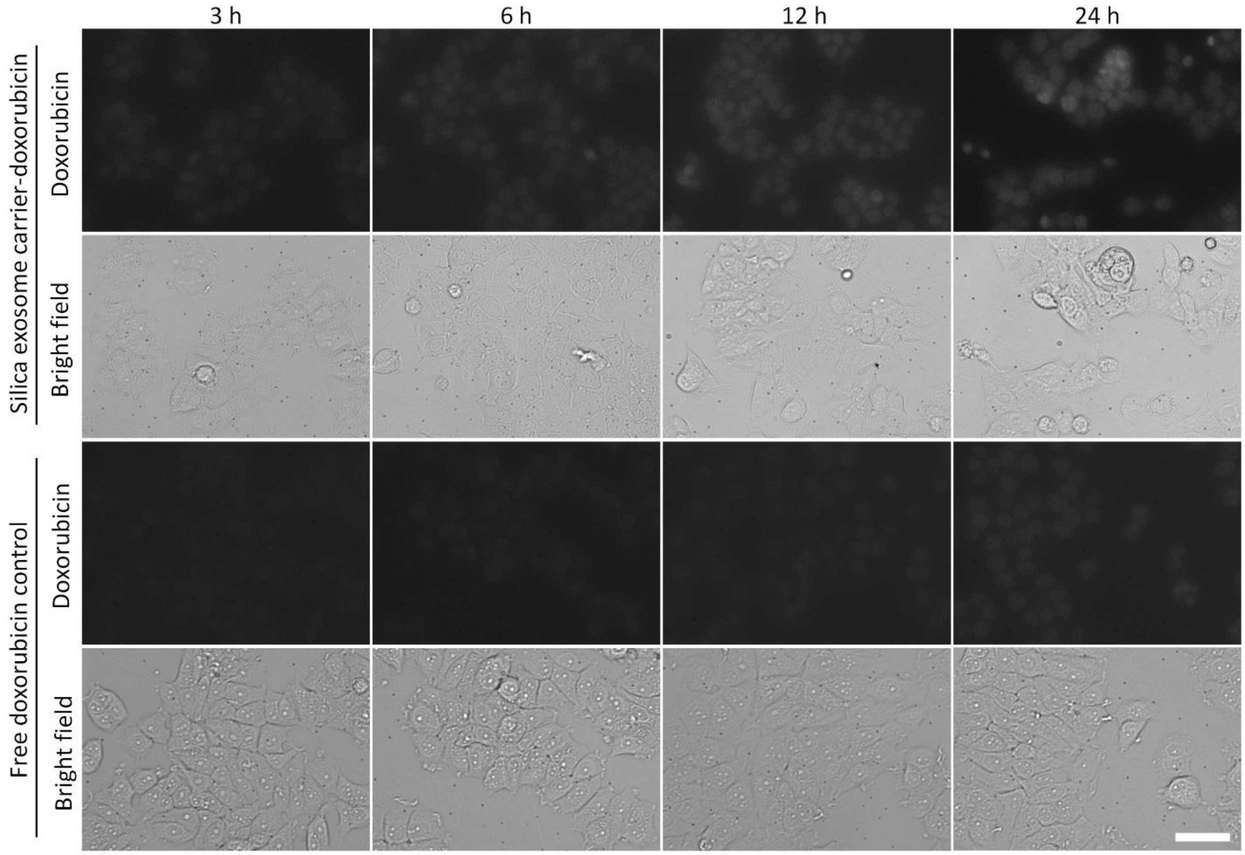

Figure S3. Microscope images of doxorubicin-loaded, 50 nm silica nanoparticles encapsulated within exosomes being intaken by MCF-7 cells. Compared to the free doxorubicin control group, doxorubicin loaded in exosome-silica nanocarriers through acoustofluidic loading demonstrated stronger doxorubicin fluorescence inside of cells. The images show higher intracellular doxorubicin fluorescence than for free doxorubicin. Thus, a higher intake efficiency for the doxorubicin-loaded and encapsulated nanoparticles is achieved. Scale bar: 50 µm.

5. Acoustofluidics derived exosome encapsulation with drug loading showing an enhanced doxorubicin intake efficiency and inhibited cell proliferation.

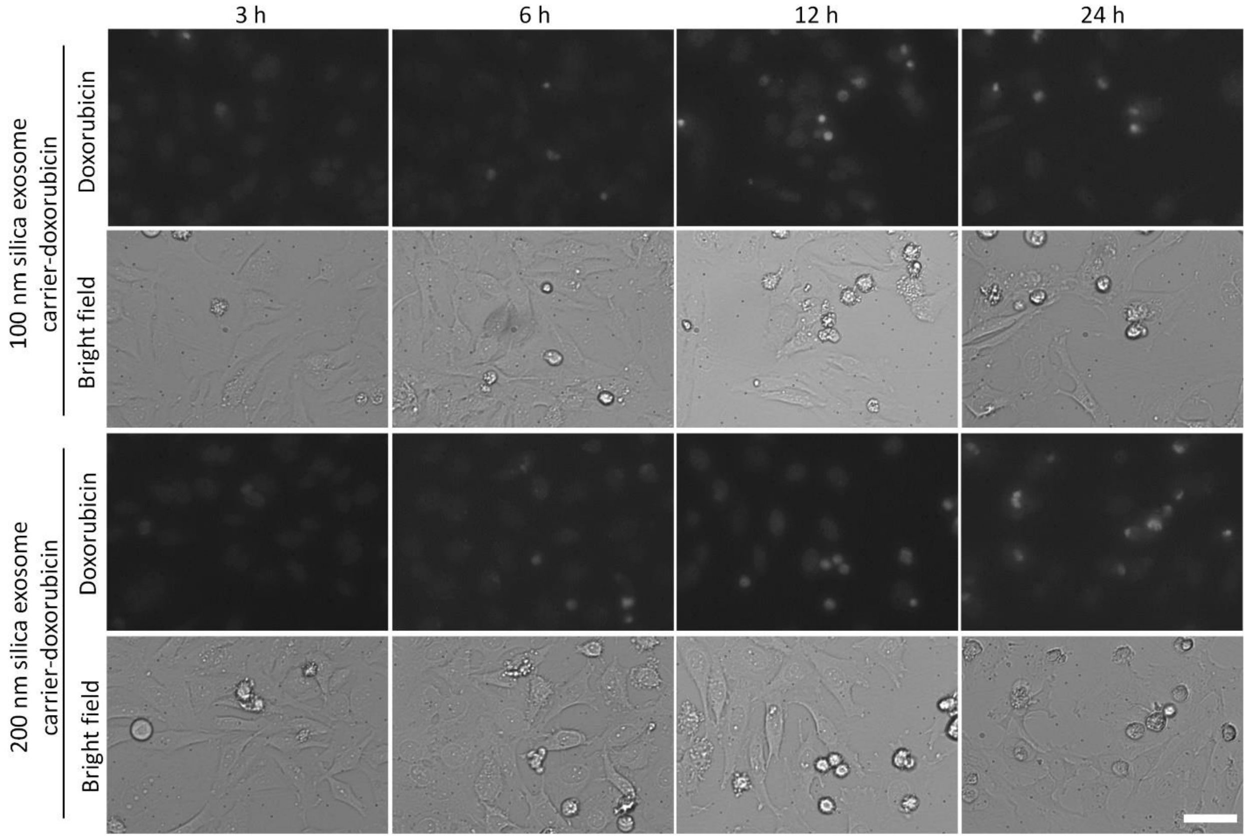

Figure S4. Acoustofluidics derived exosome encapsulation with drug loading displaying enhanced doxorubicin intake efficiency and inhibited HeLa cell proliferation. The microscope images show HeLa cell intake of doxorubicin-loaded in 100 nm spherical and 200 nm rod-shaped silica nanoparticles; the nanoparticles encapsulated by exosomes demonstrate higher intracellular doxorubicin fluorescence. Both groups exhibit increased doxorubicin fluorescence in comparison to the control groups using free doxorubicin in Figure 4A. This indicates acoustofluidic derived exosome-silica nanocarriers consisting of 100 nm and 200 nm silica nanoparticles can also enhance drug delivery efficiency for HeLa cells. Scale bar: 50 µm.

6. Acoustofluidics derived exosome encapsulation with drug loading shows an enhanced doxorubicin intake efficiency and inhibited cell proliferation.

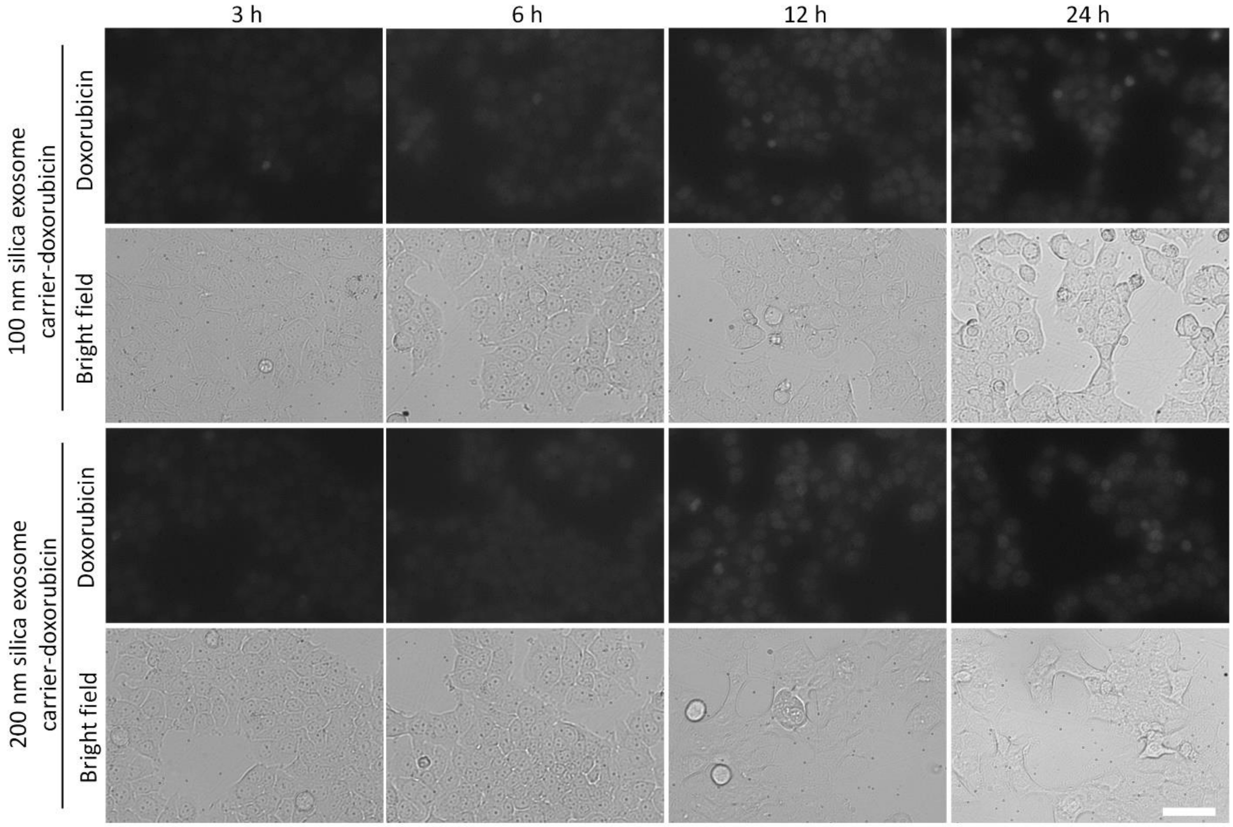

Figure S5. Acoustofluidics derived exosome encapsulation with drug loading show an enhanced doxorubicin intake efficiency and inhibited MCF-7 cell proliferation. Using MCF-7 cell intake microscopy, doxorubicin loaded in 100 nm spherical and 200 nm rod-shaped silica nanoparticles and encapsulated by exosomes show a higher intracellular doxorubicin fluorescence. Both groups show increased doxorubicin fluorescence when compared with the control groups using free doxorubicin in Figure 4A. This indicates acoustofluidic derived exosome-silica nanocarriers consisting of 100 nm and 200 nm silica nanoparticles also can enhance drug delivery efficiency for MCF-7 cells. Scale bar: 50 µm.

References

1 Ashgriz, N. *Handbook of atomization and sprays: theory and applications*. (Springer Science & Business Media, 2011).

2 Hill, R. & Eaves, L. Nonaxisymmetric shapes of a magnetically levitated and spinning water droplet. *Phys. Rev. Lett.* **101**, 234501 (2008).

3 Gu, Y. *et al.* Acoustofluidic centrifuge for nanoparticle enrichment and separation. *Sci. Adv.* **7**, eabc0467 (2021).

4 Shilton, R., Tan, M. K., Yeo, L. Y. & Friend, J. R. Particle concentration and mixing in microdrops driven by focused surface acoustic waves. *Journal of Applied Physics* **104**, 014910 (2008).

5 Lighthill, J. Acoustic streaming. *Journal of sound and vibration* **61**, 391-418 (1978).

6 Alghane, M. *et al.* Experimental and numerical investigation of acoustic streaming excited by using a surface acoustic wave device on a 128° YX-LiNbO3 substrate. *J. Micromech. Microeng.* **21**, 015005 (2010).

7 Martel, J. M. & Toner, M. Inertial focusing in microfluidics. *Annual review of biomedical engineering* **16**, 371-396 (2014).

8 Wang, Z. *et al.* Cell lysis via acoustically oscillating sharp edges. *Lab Chip* **19**, 4021-4032 (2019).

9 Nama, N., Huang, P.-H., Huang, T. J. & Costanzo, F. Investigation of micromixing by acoustically oscillated sharp-edges. *Biomicrofluidics* **10**, 024124, doi:10.1063/1.4946875 (2016).

10 COMSOL, A. Comsol multiphysics® v. 5.4 www. comsol. com. Stockholm, Sweden. *COMSOL AB* (2018).
